# The Impact of Drought Stress on Antioxidant Responses and Accumulation of Flavonolignans in Milk Thistle (*Silybum marianum* (L.) Gaertn)

**DOI:** 10.3390/plants8120611

**Published:** 2019-12-16

**Authors:** Abdelaleim I. ElSayed, Mohamed A. M. El-hamahmy, Mohammed S. Rafudeen, Azza H. Mohamed, Ahmad A. Omar

**Affiliations:** 1 Biochemistry Department, Faculty of Agriculture, Zagazig University, Zagazig 44519, Egypt; 2 Department of Agricultural Botany, Faculty of Agriculture, Suez Canal University, Ismailia 41522, Egypt; 3 Department of Molecular and Cell Biology, University of Cape Town, Private Bag, Rondebosch 7701, South Africa; Suhail.Rafudeen@uct.ac.za; 4 Agricultural Chemistry Department, Faculty of Agricultural, Mansoura University, Mansoura 35516, Egypt; azza@mans.edu.eg; 5 Citrus Research and Education Center, University of Florida, IFAS, Lake Alfred, FL 33850, USA

**Keywords:** antioxidant defense, chalcone synthase genes, reactive oxygen species, silymarin, abiotic stress

## Abstract

Biosynthesis and accumulation of flavonolignans in plants are influenced by different environmental conditions. Biosynthesis and accumulation of silymarin in milk thistle (*Silybum marianum* L.) were studied under drought stress with respect to the antioxidant defense system at the physiological and gene expression level. The results revealed a reduction in leaf chlorophyll, ascorbic acid, and glutathione contents. In contrast, H_2_O_2_, proline, and antioxidative enzyme activities, such as superoxide dismutase (SOD), catalase (CAT), peroxidase (POD), ascorbate peroxidase (APX), and glutathione reductase (GR), were increased. These results confirmed that milk thistle undergoes oxidative stress under drought stress. Furthermore, transcription levels of *APX*, *SOD*, *CAT*, *1-Cys-Prx*, and *PrxQ* were significantly increased in milk thistle under drought stress. Overall this suggests that protection against reactive oxygen species and peroxidation reactions in milk thistle are provided by enzymatic and non-enzymatic antioxidants. Flavonolignans from milk thistle seeds after different drought treatments were quantified by high-performance liquid chromatography (HPLC) and showed that severe drought stress enhanced the accumulation of silymarin and its components compared with seeds from the control (100% water capacity). Silybin is the major silymarin component and the most bioactive ingredient of the milk thistle extract. Silybin accumulation was the highest among all silymarin components in seeds obtained from drought-stressed plants. The expression of the chalcone synthase (*CHS*) genes (*CHS1*, *CHS2*, and *CHS3*), which are associated with the silybin biosynthetic pathway, was also increased during drought stress. These results indicated that milk thistle exhibits tolerance to drought stress and that seed derived from severe drought-stressed plants had higher levels of silymarin.

## 1. Introduction

Milk thistle (*Silybum marianum* (L.) Gaertn, Asteraceae) is an annual herbaceous plant, which is widespread in temperate and Mediterranean climatic regions [1]. Silymarin is the bioactive compound in milk thistle seeds, which is an isomeric mixture of flavonolignans, including silybin, isosilybin, silydianin, taxifolin, and silychristin [2]. Silymarin has been used as hepatoprotective and also inhibits or reduces the risk of emerging certain cancers [3]. The main active component of silymarin is attributed to silybin, which comprises 60%–70% of silymarin [4,5] and has been linked to the hepatoprotective properties and anti-carcinogenic capacity [6]. Biosynthesis and accumulation of bioactive compounds in plant tissues depends on environmental conditions [7,8].

Environmental stresses alter the synthesis and concentration of active substances in plants by affecting the metabolic pathways of secondary metabolites [9]. Selmar and Kleinwächter [8] reported that the concentrations of secondary plant metabolites were remarkably improved under drought stress conditions, mainly phenolic and flavonoid compounds. Secondary metabolites play an important role in plant defense mechanisms, and moreover, it is an important source of bioactive compounds that possess pharmacological activities against many human diseases. Moreover, flavonoids not only have antioxidant activities by the process of scavenging the free radicals but also modulate cell signaling pathways in plant defense against environmental stress. It has been reported that water deficiency stress may alter plant behavior by affecting plant metabolism, and therefore, drought stress has a great impact on crop production. Therefore, exposing plants to some degree of water deficit has been proposed as a potential approach to improve the production of these active constituents [10]. Moreover, drought stress results in the generation of reactive oxygen species (ROS), which have negative consequences at a cellular level: damage of membrane, DNA, lipids, and amino acids and limiting the activities of several enzymes [11]. To minimize these damaging effects, plants activate antioxidative guard systems involving both enzymatic and non-enzymatic antioxidants. The enzymatic antioxidant system that regulates ROS and redox homeostasis includes superoxide dismutase (SOD) [12], glutathione peroxidases (GPXs), peroxiredoxins (Prxs), ascorbate peroxidase (APX), and catalase (CAT) [13]. The non-enzymatic antioxidants are very important because they include phenolics and flavonoids, which not only defend plants from excessive ROS damage [14] but also certain of these metabolites have specific medicinal properties that have been shown to be useful in the treatment of various human diseases and illnesses [14].

Recently, Zahir et al. [15] stated that drought stress boosted the accumulation of the total phenolic and flavonoid substances in milk thistle plants. Biosynthesis of flavonoids in milk thistle and other plants depend on a critical enzyme, chalcone synthase (CHS) [16,17]. The activities of CHSs genes were found to be regulated by endogenous mechanisms in plant development [18,19] or by diverse exogenous stimuli. These included low temperature [20], gamma irradiation and salinity treatments [21], and methyl jasmonate and salicylic acid [22]. Though drought stress can stimulate and enhance the accumulation of secondary plant metabolites, it also affects the growth of the plant at the physiological and biochemical levels. To date, there have been very few reports on the effect of water deficit on the accumulation and biosynthesis of silymarin in milk thistle seeds. Therefore, the present study aimed to investigate the impact of drought stress on the accumulation of silymarin in seeds and regulation of biosynthetic pathways related to silymarin synthesis. Furthermore, the antioxidant defense system in response to drought stress at the physiological and transcriptional levels was also investigated.

## 2. Results

### 2.1. Growth Rate, Total Chlorophyll, and Proline Content

**Figure 1:**
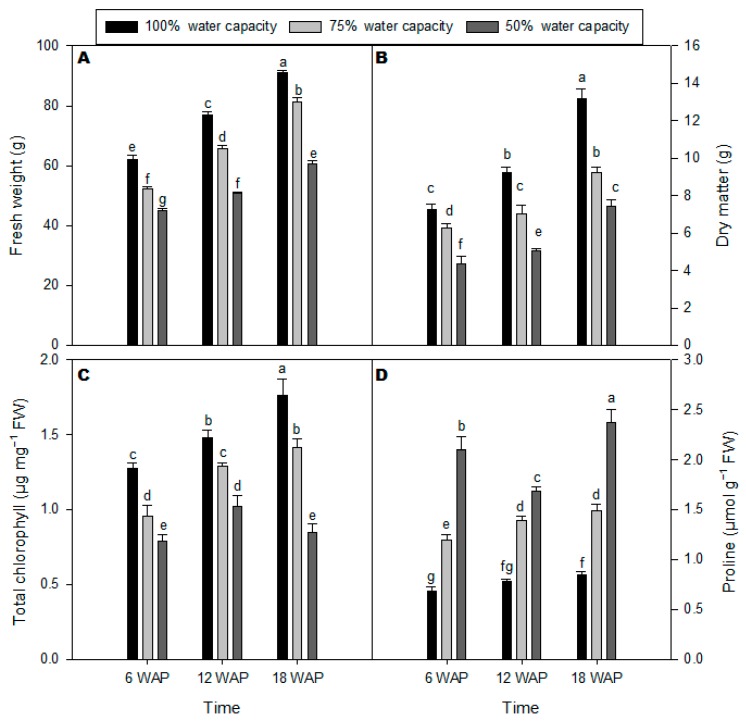
Effects of different water-deficit stress (100% water capacity, 75% water capacity, and 50% water capacity) on (**A**) Fresh weight; (**B**) Dry mass; (**C**) Total chlorophyll content (μg·mg^−1^ DM); and (**D**) Proline contents (µmol·mg^−1^ DM) of milk thistle at 6, 12, and 18 weeks after planting (WAP). Means ± SDs, *n* = 3 from three independent experiments. Different letters above bars within treatment groups are significantly different (*p* < 0.05). FW = fresh weight and DM = dry matter.

A drought stress effect on the growth of milk thistle was evident at 75% water capacity, as indicated by the significant reduction of fresh weight (FW) and dry matter (DM) at 50% water capacity treatment compared with the well-watered plants (Figure 1A,B). Total chlorophyll content of milk thistle leaves decreased under drought stress at 50% and 75% water capacity treatments (Figure 1C). The differences in chlorophyll content were observed across all time points after planting (Figure 1C). There was a differential accumulation of proline in milk thistle in response to drought stress treatments at different time points after planting (Figure 1D). There was a marked increase in proline levels in severe drought-stressed milk thistle plants (50% water capacity) 18 weeks after planting (WAP) compared with other time points, particularly the well-watered control plants. However, the lowest proline content in milk thistle at 50% water capacity was at 12 WAP (Figure 1D).

### 2.2. Malondialdehyde, Ascorbic Acid, Glutathione, and H_2_O_2_ Contents

**Figure 2:**
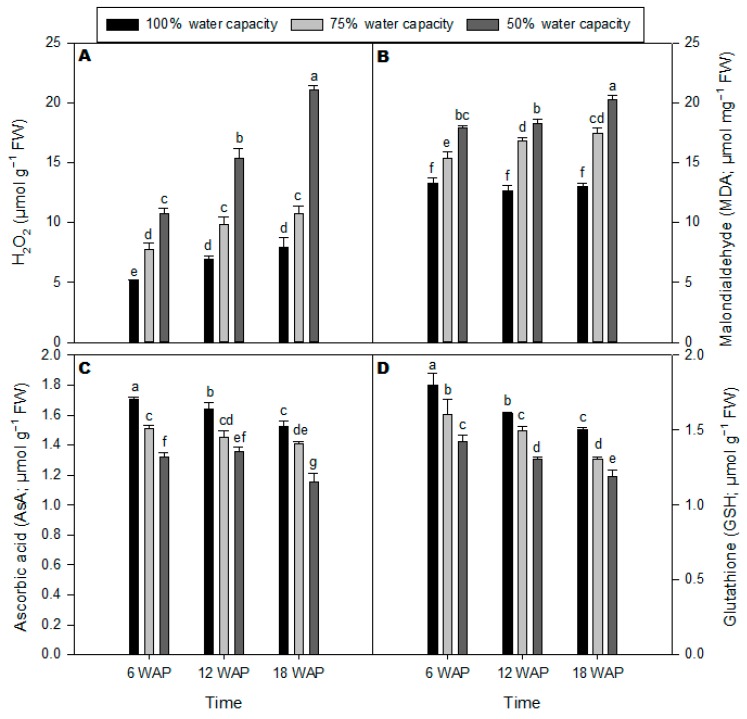
Malondialdehyde, ascorbic acid, glutathione, and H_2_O_2_ contents. (**A**) H_2_O_2_ (µmol·mg^−1^ DM); (**B**) Lipid peroxidation (malionaldehyde equivalents; μmol·g^−1^ DM); (**C**) Ascorbic acid (AsA; μmol·mg^−1^ DM); (**D**) Glutathione (GSH; μmol·mg^−1^ DM) of milk thistle under different water-deficit stress (100% water capacity, 75% water capacity, and 50% water capacity) at 6, 12, and 18 weeks after planting (WAP). Means ± SDs, *n* = 3 from three independent experiments. Different letters above bars within treatment groups are significantly different (*p* < 0.05).

The H_2_O_2_ content markedly increased in milk thistle leaves under drought stress at 75% and 50% water capacity, respectively, and across all time points after planting (Figure 2A). The highest and most significant amount of H_2_O_2_ was observed at 50% water capacity 18 weeks after planting (Figure 2A). Lipid peroxidation levels in milk thistle leaves were measured and calculated based on malondialdehyde (MDA) concentration (Figure 2B). MDA content increased considerably under drought stress, particularly at 18 WAP. There were no significant differences in MDA content for well-watered plants across all time points. The concentration of ascorbic acid (AsA) in milk thistle leaves was significantly reduced under drought stress at 50% water capacity across all time points after planting compared with control plants (Figure 2C). The lowest amount of AsA was observed at 18 WAP with severe drought stress. Similarly, glutathione (GSH) content in milk thistle leaves was significantly reduced under drought stress at 50% water capacity across all time points compared to well-watered plants (Figure 2D).

### 2.3. Antioxidant Enzyme Activities

**Figure 3:**
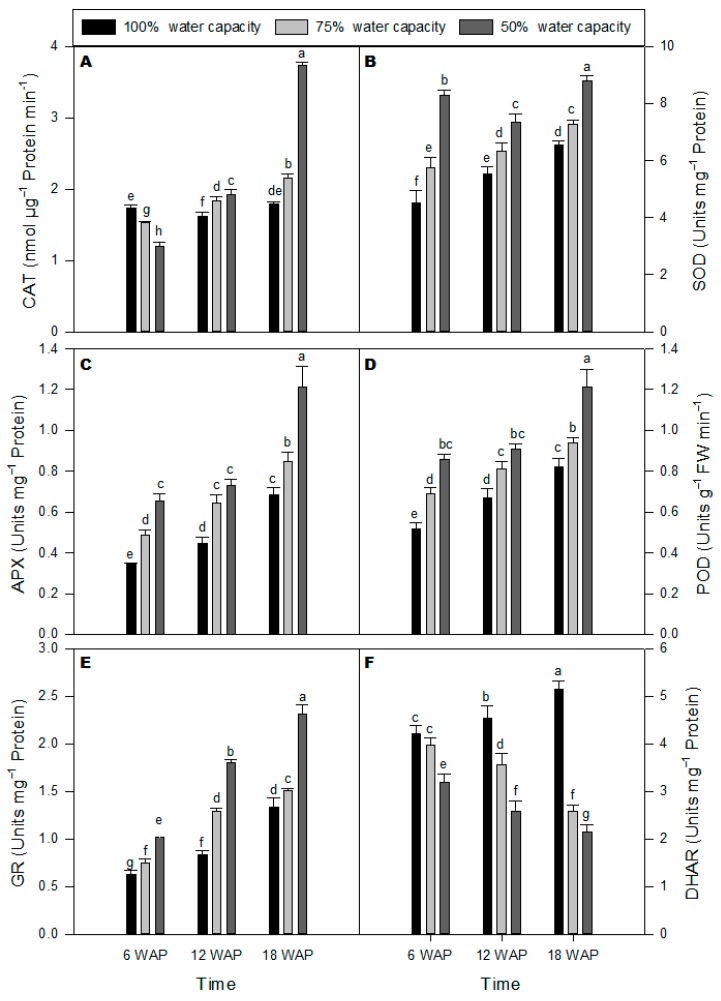
Effects of different water-deficit stress (100% water capacity, 75% water capacity and 50% water capacity) on activities of antioxidant enzymes in milk thistle at 6, 12, and 18 weeks after planting (WAP): (**A**) catalase (CAT), (**B**) superoxide dismutase (SOD), (**C**) ascorbate peroxidase (APX), (**D**) peroxidase (POD), (**E**) glutathione reductase (GR), and (**F**) dehydroascorbate reductase (DHAR). Means ± SDs, *n* = 3 from three independent experiments. Different letters above bars within treatment groups are significantly different (*p* < 0.05).

Enzyme (superoxide dismutase (SOD), catalase (CAT), peroxidase (POD), ascorbate peroxidase (APX), glutathione reductase (GR), and dehydroascorbate reductase (DHAR)) activities were measured in milk thistle leaves under drought stress at different water capacity treatments compared with well-watered plants (Figure 3). CAT activity decreased at 50% water capacity at six WAP but subsequently increased at 12 and 18 WAP at the same water capacity when compared with the control (Figure 3A). The maximum CAT activity was detected with 50% water capacity 18 WAP (Figure 3A). There was a significant increase in SOD activity across all time points and at both 75% and 50% water capacity compared to the respective controls (Figure 3B). The highest increase in SOD activity was observed for drought-stressed plants treated at 50% water capacity 18 WAP. APX activity increased across all time points for both drought stress treatments (Figure 3C). APX activity was significantly increased at 50% water capacity 18 WAP compared with the well-watered plants. Similarly, POD activity increased overtime for all time points and drought treatments (Figure 3D). The highest POD activity was observed with 50% water capacity treatment 18 WAP (Figure 3D). GR activity increased significantly under severe drought (50% water capacity) across all time points with the highest activity observed 18 WAP (Figure 3E). DHAR activity under severe drought stress (50% water capacity) was considerably reduced when compared with the well-watered plants with the lowest DHAR activity observed 18 WAP (Figure 3F).

### 2.4. Transcription Levels of Genes Encoding Antioxidant Enzymes

**Figure 4:**
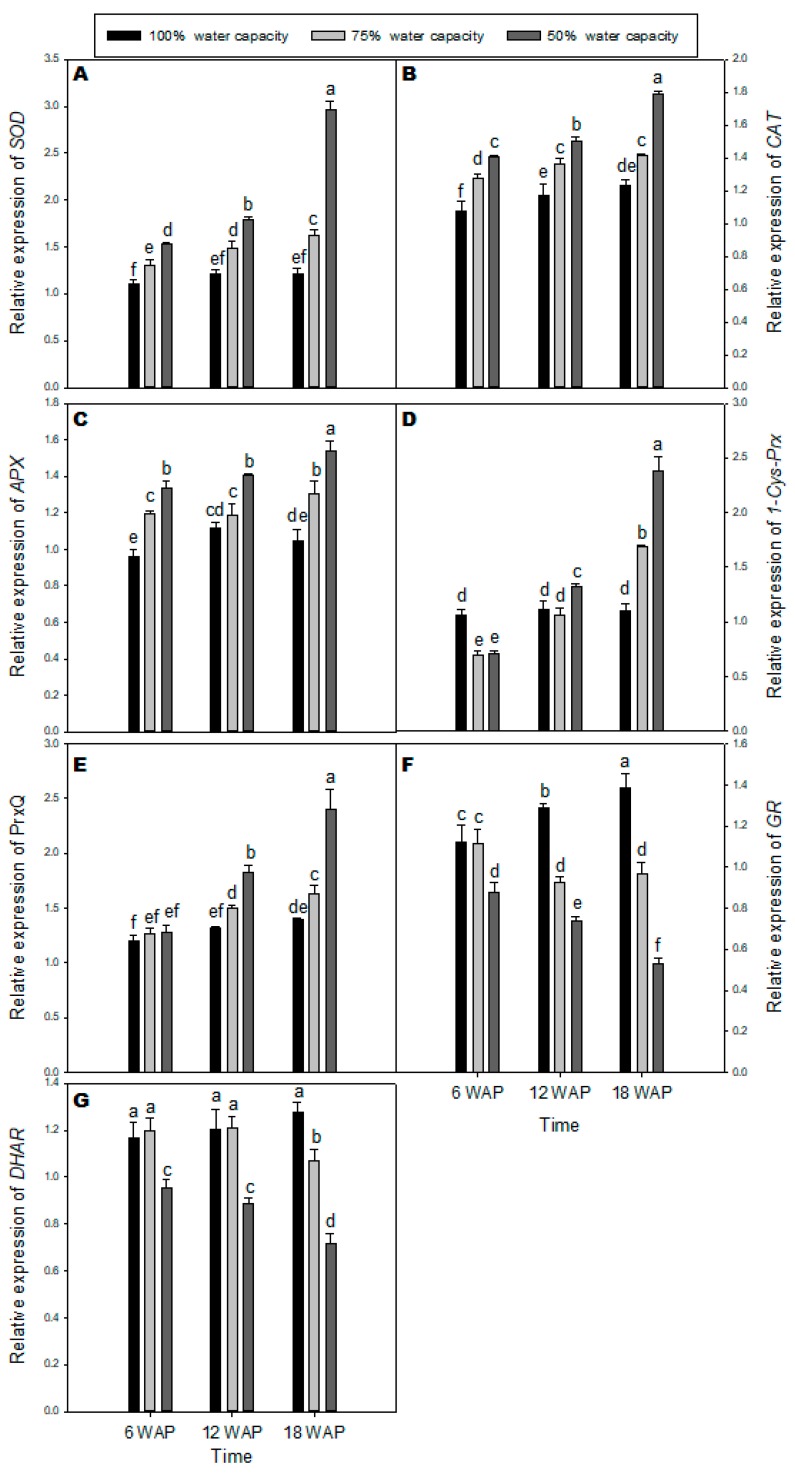
Transcript levels of antioxidant enzymes encoding genes: (**A**) *SOD*, (**B**) *CAT*, (**C**) A*PX*, (**D**) *1-Cys-Prx*, (**E**) *PrxQ*, (**F**) *GR*, and (**G**) *DHAR* in milk thistle under different water-deficit stress (100% water capacity, 75% water capacity, and 50% water capacity) at 6, 12, and 18 weeks after planting (WAP). Transcript amounts were quantified by qPCR and relative to actin transcript levels. Means ± SDs, *n* = 3 from three independent experiments. Different letters above bars within treatment groups are significantly different (*p* < 0.05).

Real Time PCR (qPCR) was used to study the expression level of different genes encoding antioxidant enzymes in drought-stressed milk thistle compared with the well-watered plants. The *SOD* transcript level slightly increased under drought stress at 6 and 12 WAP in milk thistle leaves compared with the well-watered plants (Figure 4A). The uppermost increase in the *SOD* transcript level was observed with severe drought 18 WAP. The expression of *CAT* increased slightly but significantly under drought stress in milk thistle leaves across all time points with the highest level detected 18 WAP at 50% water capacity (Figure 4B). The transcript level of *APX* increased in drought-stressed milk thistle leaves across all time points with significantly higher levels at severe drought stress 18 WAP compared with the well-watered plants (Figure 4C). The level of *1-Cys-Prx* transcript decreased at six WAP for drought stress at 75% and 50% water capacity, respectively (Figure 4D). Transcript levels of *1-Cys-Prx* significantly increased under mild and severe drought stress at 18 WAP compared with the well-watered plants. There were no significant differences in expression levels of *PrxQ* at six WAP under drought stress when compared to the control (Figure 4E). Nevertheless, *PrxQ* transcript showed a significant increase under severe drought stress at 12 and 18 WAP compared with the well-watered plants. The transcript level of *GR* was significantly decreased under drought stress at 50% water capacity compared with the well-watered plants across all time points (Figure 4F). The lowest amount of *GR* transcript was found at 18 WAP under severe drought stress. There was no significant difference in *DHAR* transcript level at mild drought stress (75% water capacity) and the well-watered plants at 6 and 12 WAP (Figure 4G). Conversely, there was a significant decrease in *DHAR* expression level under severe drought stress (50% water capacity) for all time points compared with other treatments (Figure 4G).

### 2.5. Transcript Levels of CHS1, CHS2, and CHS3 Genes

**Figure 5:**
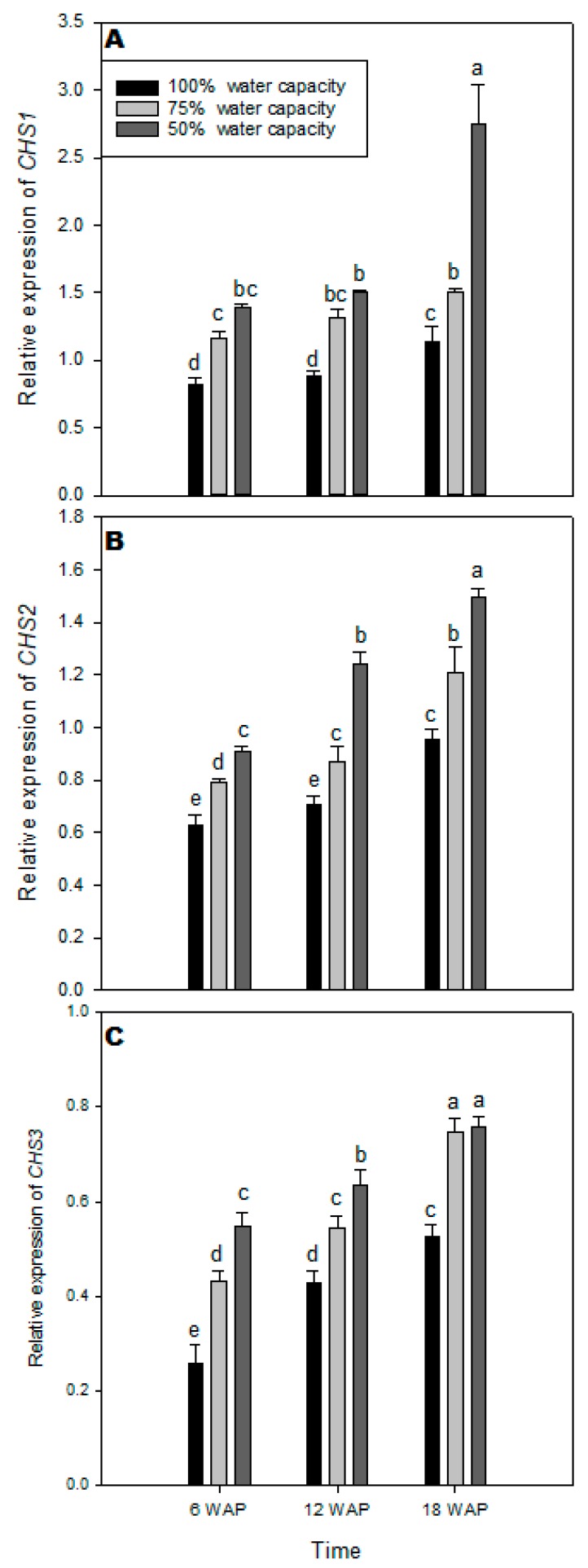
Transcript levels of chalcone synthase genes (*CHS1*, *CHS2*, and *CHS3*) of milk thistle, showing different responses under different water-deficit stress (100% water capacity, 75% water capacity, and 50% water capacity) at 6, 12, and 18 weeks after planting (WAP). Transcript amounts were quantified by qPCR and relative to actin transcript levels. Means ± SDs, *n* = 3 from three independent experiments. Different letters above bars within treatment groups are significantly different (*p* < 0.05).

Quantitative RT–PCR was used to analyze the relative transcript levels of three genes involved in the flavonolignan biosynthesis in milk thistle [17], namely chalcone synthase genes (*CHS1*, *CHS2*, and *CHS3*). The transcript levels of *CHS1* increased in milk thistle leaves exposed to drought stress compared with the well-watered plants (Figure 5A). Drought treatment with 50% water capacity resulted in the highest increase in the transcript level of *CHS1* 18 WAP compared with the well-watered plants (Figure 5A). The transcript level of *CHS2* increased in milk thistle leaves across all time points under drought stress plants compared with the well-watered plants (Figure 5B). There were significantly higher levels of *CHS2* transcript 18 WAP under drought stress at 50% water capacity. There was an increase in *CHS3* transcript under drought stress across all time points when compared with the control (Figure 5C). However, at 18 WAP, there were no significant differences in transcript levels of *CHS3* gene under drought treatments at 75% and 50% water capacity (Figure 5C).

### 2.6. Flavonolignans Contents in Dried Seeds of Milk Thistle

Changes in the flavonolignans in milk thistle seeds were quantified under drought stress and well-watered conditions (Table 1). Milk thistle grew under mild (75%), and severe (50%) drought stress conditions contained the highest amount of silymarin, 19.91 mg·g^−1^ dry matter (DM) and 23.76 mg·g^−1^ DM, respectively, compared with seeds from plants grown under well-watered conditions which included 18.70 mg·g^−1^ DM (Table 1). Data obtained by HPLC analysis revealed that silybin was the main flavonoligan in milk thistle seeds, which was significantly increased (13.87 mg·g^−1^ DM) under severe drought stress compared to mild drought stress (12.34 mg·g^−1^ DM) and well-watered plants (11.44 mg·g^−1^ DM). The components of silymarin in seeds obtained from drought-stressed milk thistle consisted of silybin (58.3%), isosilybin (18%), silydianin (10.3%), silychristin (7.3%), and taxifolin (5.8%) (Table 1).

### 2.7. Relatedness of Chalcone Synthase (CHS1, CHS2, and CHS3) Sequences.

**Figure 6:**
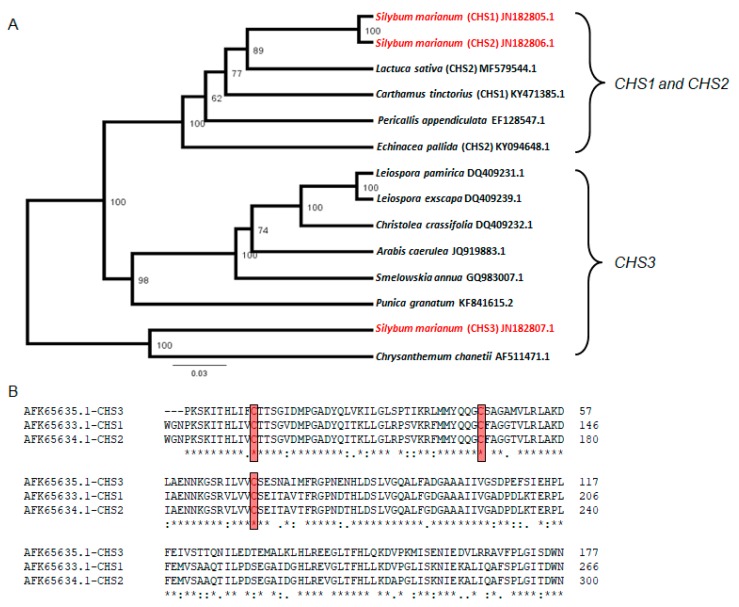
(**A**) Dendrogram depicting phylogenetic relationships among 14 different chalcone synthase isoforms. The numbers above the branches indicate the bootstrap confidence value. The scale bar shows the number of substitutions per amino acid. Bootstrap analysis of 500 replicates was performed. (**B**) Alignment of partial sequences of the deduced amino acid sequences of chalcone synthase genes (*CHS1*, *CHS2*, and *CHS3*) for milk thistle available in the GenBank databases based on the amino sequences. The red box indicates cysteine conservation. In the lowest line ‘*’ indicates full conservation among all sequences, ‘:’ shows highly conservative exchanges and ‘.’ indicates conservative exchanges of amino acids.

Multiple alignments of full-length cDNA sequences of *CHS1*, *CHS2,* and *CHS3* genes from milk thistle and various plants were used to construct a phylogenetic tree using the unweighted pair group method with arithmetic mean (UPGMA) method. The phylogenetic tree showed that these cDNA sequences segregated into two major groups (Figure 6A). Group I contained CHS1 and *CHS2* genes, which had CHS1 and *CHS2* genes from milk thistle with 100% identity (Figure 6A). *CHS3* from milk thistle was separated in different groups, which shared 100% identity with CHS from Chrysanthemum chanetii. Alignment of deduced amino acid sequences of *CHS1*, *CHS2*, and *CHS3* from milk thistle plants using CLUSTALW 2.1 (Science Foundation Ireland, Upper, Dublin 2, Ireland) indicated that cysteine was highly conserved in chalcone synthase (Figure 6B). The identities between *CHS1* and *CHS2* were high (100%), but *CHS3* isoform share low similarity with them (80%).

## 3. Discussion

*Silybum marianum* (L.) Gaertn (milk thistle, Asteraceae) is a medicinally important plant whose secondary metabolites have been extensively studied at the level of their chemistry, bioactivity, and formulation in natural products [3]. However, there has been very little research done on understanding the mechanisms of how milk thistle responds to environmental stresses, such as drought stress, and the associated increased accumulation and biosynthesis of silymarin. The present study, therefore, aimed to understand the effect of water deficit on the accumulation of silymarin in milk thistle seeds in the context of the antioxidant defense system at the physiological and transcriptional level. Drought stress significantly affected the growth rates of milk thistle with fresh and dry weights of milk thistle negatively affected by drought stress compared to well-watered plants (Figure 1A,B). The obtained results from the current study showed that chlorophyll content in milk thistle leaves was also reduced (Figure 1C) by drought stress with the reduction of chlorophyll content as a result of either suppression of chlorophyll biosynthesis or breakdown. This observation has been contemplated as a distinctive symptom of oxidative stress [23].

Furthermore, oxidative stress causes impairment in chloroplast structure, which can also lead to a reduction in chlorophyll content in plants [24] and subsequently reduces the plant growth. The accumulation of proline and soluble sugars under drought stress and their role in drought tolerance are well-known in many plant species [25]. In this study, proline content increased sharply in milk thistle leaves under severe drought stress compared to moderate drought stress and the well-watered plants, especially at 6 and 18 WAP (Figure 1D). Several studies reported that the accumulation of proline content was remarkably higher in drought-tolerant cultivars than drought-sensitive cultivars of olive [26], mulberry [27], and wheat [28]. Accumulation of the osmolyte, proline, under drought stress, guards plant cells by allowing osmotic adjustment in the cytosol and vacuole to that of the external environment [29,30].

In this study, AsA and GSH contents decreased in milk thistle leaves under severe drought stress conditions (Figure 2C,D). However, reduced GSH has pivotal roles in plants, involving antioxidant defense and redox signaling [31] and regulating gene expression in plant cells [32]. GSH acts as an antioxidant that prevents excessive oxidation in the cell and directly engages in the reduction of most active oxygen radicals produced due to stress [31]. Furthermore, Mhamdi et al. [33] stated that ascorbate levels could continue to be highly reduced if there are sufficient amounts of GSH and/or other thiols presented. It appears that the non-enzymatic antioxidants (AsA and GSH) may have decreased as a result of scavenging H_2_O_2_, which had accumulated rapidly in milk thistle leaves under water deficit stress. Lipid peroxidation of biological membranes often leads to structural adjustments in the plants grown under drought stress conditions. MDA content commonly used to evaluate the lipid peroxidation level resulting from oxidative stress [23]. In the present study, increasing MDA substance was accompanied by water deficit stress (Figure 2B), which indicated that drought stress-induced membrane lipid peroxidation resulting in membrane fluidity and leading to improved membrane permeability [27]. Higher amounts of either constitutive or induced antioxidants empower plants to have better tolerance to oxidative damage [34].

Different ROS produced in plant cells [35] are controlled by different antioxidants and enzymatic and non-enzymatic antioxidant defense pathways. The enzymatic antioxidants pathway include CAT, SOD, APX, POD, GR, and DHAR [36]. In the current study, CAT activity in milk thistle grew under drought stress was significantly higher at 18 WAP. Similar trends were observed for SOD activity, which increased under drought stress across the time points sampled (Figure 3B). Superoxide radicals, such as H_2_O_2_, are potent oxidants, and their damaging effects are prevented by the ascorbate–glutathione cycle [35]. The reactive and toxic oxide, OH^−^ reacts with all macro-molecules, but its action is prevented or reduced by SOD and CAT [34]. In the current work, APX, POD, and GR activities increased under drought stress due to their role in scavenging and consuming H_2_O_2_ and other ROSs generated by drought stress. The obtained results indicated that the milk thistle plant controlled the damage from oxidative stress by using an enzymatic antioxidant defense system. High activities of antioxidant enzymes have also been observed in drought-resistant plants from many genera and species [26,27,37,38,39]. SODs are considered the first-line defense in the water-water cycle of the antioxidant system [40] and play a crucial role in quenching ROS [41]. SODs catalyze the dismutation of O_2_^−^ into H_2_O_2,_ which are then excluded by antioxidant enzymes, such as CAT, APX, and POD. There appears to be a link between the SOD activity and the activities of APX, CAT, and POD in the current study, as suggested by the increase in SOD activity with accompanying increases in CAT, APX, and POD [42]. An increase in the content of proline, together with the antioxidant enzyme activities (CAT, SOD, APX, and POD) were also detected in the present study. Proline accumulation in plant cells could trigger the antioxidant defense systems [26,38] and also steady the structure and activities of enzymes [43]. It is, therefore, possible that the high content of proline in milk thistle leaves under extended severe water deficit may also promote the high activities of the antioxidant enzymes.

Overall, the results of qRT-PCR showed that prolonged severe drought stress of milk thistle increased gene expression of *APX*, *SOD,* and *CAT* compared with plants grown under well-watered conditions. In contrast, expression of *DHAR* and *GR* in drought-stressed milk thistle plants were inhibited. However, DHAR and GR are critical enzymes involved in the ascorbate glutathione cycle and play essential roles in ROS detoxification [44]. To obtain additional insight into the mechanisms of drought tolerance in the milk thistle, the gene expression of peroxiredoxins was studied. The expression of *1-CysPrx* and *PrxQ* was abundant in drought-stressed milk thistle plants compared with those grown under well-watered conditions. Lee et al. [45] reported that transgenic tobacco plants expressing the rice *1-CysPrx* gene displayed more tolerance to oxidative stress when compared with the control, suggesting that the principal role of *1-CysPrx* is protection against oxidants. Furthermore, *PrxQ* is presumed to be chloroplastic and detoxifies alkyl hydroperoxides [46]. Under drought stress, the high expressions of *PrxQ* and *1-Cys-Prx* in milk thistle plants suggest that these genes are involved in antioxidant defense and redox signaling. Hossain et al. [47] reported that *1-Cys-Prx* protects the chloroplast structures against oxidative damage by contributing to detoxification processes.

The amount of phenolic acids and flavonoids in milk thistle has been increased under drought stress [15,48]. Flavonoids possess antioxidant properties related to their ability to scavenge free radicals, and they also modulate cell signaling pathways. The results obtained from the current study showed that plants grown under mild and severe drought treatments contained silymarin levels more significant than those grown under well-watered conditions (Table 1). Comparable to the results of the study, Afshar, Chaichi, Jovini, Jahanzad, and Hashemi [48] also stated silymarin in milk thistle seeds grown under drought stress had been accumulated in higher amounts compared to the control treatment. In the present study, silybin was the major flavonoligan in milk thistle seeds constituting 58.3% of the total phenolic compounds of the milk thistle seed (Table 1). It is noteworthy that taxifolin and coniferyl alcohol are the precursors of silybin, which is catalyzed by ascorbate peroxidase (APX1) (Figure S2). In this reaction, the APX1 oxidizes coniferyl alcohol to a radical that couples with equimolar of taxifolin to form silybin [49,50]. APXs are commonly considered to be detoxifiers that eradicate peroxides, such as H_2_O_2_ and O_2_^−^, by utilizing ascorbate as the electron donor [51].

It has been reported that environmental stresses have a strong impact on the accumulation of secondary metabolites in plants [7]. Flavonoids are important secondary metabolites that play a pivotal role in the plant’s defense system against biotic and abiotic stresses [52,53,54]. Furthermore, abundant accumulation of these metabolites (polyphenols) plants grown under stressful environmental conditions may prevent the excess generation of ROS and consequent damage by photoinhibition [8]. It is known that internal CO_2_ drastically declines in plant cells under drought stress due to stomatal closure causing lower utilization of NADPH and ATP, which subsequently exposes chloroplasts to excess excitation energy and finally increases ROS generation [55]. To guard cells against ROS damage by oxidative stress, ROS is scavenged by highly reduced compounds, such as phenols or flavonoids, which consume NADPH [15,56]. Maurino et al. [11] also reported that antioxidants and antioxidant-enzymes function interrupt the cascades of uncontrolled oxidation caused by ROS in plants under environmental stress conditions. Consequently, it seems that the improvement of silymarin biosynthesis in milk thistle plants is a part of the antioxidative defense mechanism system against water deficit, which helps this plant to tolerate different environments’ stressful conditions for a long time.

Chalcone synthase (CHS) is a vital enzyme of the flavonoid and flavonolignans biosynthesis pathway [17,57]. Silymarin is synthesized in milk thistle plants via the phenylpropanoid pathway converting phenylalanine to 4-coumaroyl-CoA (Figure S2). CHS and chalcone isomerase (CHI) are the enzymes that catalyze the condensation of three malonyl-CoA molecules and 4-hydroxycin-namoyl-CoA to produce the chalcone derivative, naringenin, which is the first dedicated step in the pathway of phenylpropanoid in plants [57,58]. This latter compound is hydroxylated, giving at the end taxifolin, which is the precursor of the flavonoid part of silymarin [58]. It has been shown that *CHS* gene expression is stimulated in plants under abiotic and biotic stress conditions [16]. The current study showed that the expression level of the three *CHS* genes in milk thistle leaves was highly expressed under drought stress (Figure 5A–C). The transcription level of *CHS1* was significantly higher compared with *CHS2* and *CHS3* (Figure 5). The obtained results were in agreement with previously reported studies [17,21]. Overall, our findings revealed that drought stress improved the expression of the three genes (*CHS1*, *CHS2,* and *CHS3*), which was coordinated by a definite increase in silymarin content (23.76 mg·g^−1^ DM) in milk thistle. Torres and Corchete [57] observed a relationship between the expression of *SmCHS* genes engaged in the silymarin biosynthesis pathway and the production of these metabolites in milk thistle. Sanjari, Shobbar, Ebrahimi, Hasanloo, Sadat-Noori and Tirnaz [17] identified *CHS1*, *CHS2,* and *CHS3* genes in different parts of milk thistle plant (different leaves, petals, flower heads, and diverse parts of the stem), which are perhaps involved in the silymarin biosynthetic pathway in milk thistle. In plants, the expression of *CHS* can be induced by Ultraviolet (UV), salinity, drought, wounding, irradiation, herbivory, and microbial pathogens, which subsequently enhance the production of flavonoids and silymarin in milk thistle plants [16,17,21]. Furthermore, Dao, Linthorst, and Verpoorte [16] reported that *CHS* genes play an essential role in regulating the flavonoid biosynthesis pathway, which triggers an increase in flavonoid and isoflavonoid phytoalexins and are engaged in the salicylic acid defense pathway.

Phylogenetic studies revealed that all the analyzed *CHS* genes were correlated to each other with variable distances (Figure 6A,B). The present study showed that silymarin accumulation in response to drought stress occurs in tandem with antioxidant defense responses. It will be of interest for future research to see if the signaling pathways that lead to both responses overlapping, or they are two independent pathways.

## 4. Materials and Methods 

### 4.1. Plant Material and Treatments

Milk thistle seeds [*Silybum marianum* (L.) Gaertn] were obtained from the Horticulture Department, Faculty of Agriculture, Zagazig University, Zagazig, Egypt. Seeds of milk thistle were surface sterilized by immersing in 0.1% HgCl_2_ solution for 2 min, 70% Ethanol for 3 min, and washed three times with distilled water. The seeds were then sown in plastic pots (40 cm diameter, 35 cm depth) packed with sand free from any cations or anions as a growth medium. Experiments were performed in open greenhouse conditions with 19/10 ± 2 °C as day/night temperature and relative humidity of 62%–65%. Plants were irrigated using half-strength Hoagland solution [59] (Sigma-Aldrich, Saint Louis, MO, USA). Up to full emergence, the nutrient solution was supplied at 100% water capacity every two days to all pots. Four weeks after planting (WAP), the water supply was changed at different levels, as following: 100% water capacity (well-watered), 75% (mild stress), and 50% of water capacity (severe stress). Leaf samples of milk thistle plants were collected randomly from various leaves along the shoot at early developmental stage (6 weeks), medium developmental stage (12 weeks), and a late developmental stage (18 weeks) from all the treatments under study as well as the control and stored at −80 °C before analysis for each of the measured parameters.

### 4.2. Measurement of Fresh and Dry Weight

For determination of fresh weight (FW), milk thistle was weighed after washing with sterilized distilled water, then the samples were dried at 70 °C for 72 h to determine the dry mass (DM).

### 4.3. Chlorophyll Content

Plant leaf samples at developmental stages 6, 12, and 18 WAP were harvested from different irrigation regime treatments, immediately frozen in liquid nitrogen, ground to a fine powder, and extracted with 100% acetone. Total chlorophyll (*a* and *b*) was determined spectrophotometrically, as described by Lichtenthaler [60]. 

### 4.4. Estimation of Proline Content

The proline content was assayed in milk thistle leaves, as described by Vicente et al. [61]. The samples were measured at 520 nm and calculated by using the standard curve of L-proline. Proline content in the samples was determined as micromole proline per gram of DM.

### 4.5. Determination of Malondialdehyde (MDA) Contents

The method by Rao and Sresty [62] was modified to measure MDA concentration (a product of lipid peroxidation). Frozen leaves of milk thistle (300 mg) were homogenized in 0.1% trichloroacetic acid (TCA) on ice. The homogenate was then centrifuged at 10,000× *g* for 10 min at 4 °C, and the pellet was extracted twice with the same solvent. Zero-point five millileters of supernatant was mixed with 1.5 mL of 20% TCA, inclosing 0.5% thiobarbituric acid and heated at 95 °C for 25 min, the mixture cooled to room temperature (RT), then centrifuged for 10 min at RT. The sample was measured at 532 nm and corrected by non-specific absorption at 600 nm. The MDA content was expressed as micrograms·MDA·milligram to the negative one·DM.

### 4.6. Determination of AsA and GSH Content

AsA concentrations in milk thistle leaves were calculated based on an ascorbic acid standard curve according to the method described by Mukherjee and Choudhuri [63]. The sample absorbance was measured at 530 nm. GSH content was determined, as described by Griffith [64]. Fresh leaf tissues (300 mg) were homogenized in 2 mL of 2% (*v/v*) metaphosphoric acid and centrifuged for 10 min at 18,000× *g*. Six hundred microliters of 10% (*w/v*) sodium citrate was added to 900 µL of the supernatant to neutralize the sample. One hundred microliters of the neutralized extract was added to 700 µL of 0.3 mM NADPH, 100 µL of 6 mM 5,5′-dithio-bis-2-nitrobenzoic acid, and 100 µL distilled water and was steadied at 25 °C for 3–4 min. After that, the glutathione reductase (GR) (10 µL of 50 Units) was added to the sample. The absorbance was detected at 412 nm to determine GSH levels from a standard curve.

### 4.7. Determination of H_2_O_2_ Content

Hydrogen peroxide (H_2_O_2_) concentration was assayed according to the method of Velikova et al. [65]. Leaf samples of milk thistle (300 mg) were homogenized under cooled conditions with 3 mL of 0.1% (*w/v*) TCA. Then the mixture was centrifuged for 15 min at 21,000× *g,* and 500 µL of the supernatant was mixed with 500 µL of 10 mM Potassium phosphate buffer, pH 7, and 1 mL of 1 M potassium iodate. The absorbance was measured at 390 nm. The concentration of H_2_O_2_ was estimated based on a standard calibration curve, which made by using various concentrations of H_2_O_2_.

### 4.8. Determination of Antioxidant Enzymes Activities

Leaf samples of milk thistle plants (500 mg) were frozen in liquid nitrogen, ground to a fine powder, and extracted with 10 mL of 50 mM phosphate buffer, pH 7. The extraction solution was centrifuged at 21,000× *g* for 30 min at 4 °C. The supernatant was then filtered and used to determine different antioxidant enzyme activities. Total soluble protein content was determined by using the Bradford method [66]. SOD activity was determined according to the method of Giannopolitis and Ries [67] by examining SOD-mediated inhibition of the photochemical reduction of nitro blue tetrazolium (NBT). SOD activity (one unit) was defined as the amount of enzyme required for 50% inhibition of the reduction of NBT, as measured at 560 nm. CAT activity was determined as described by Aebi [68]. The assay mixture contained 100 µL of the enzyme extract, 0.1 mM phosphate buffer pH 7, 0.1 mM Ethylenediaminetetraacetic acid (EDTA), and 0.3% H_2_O_2_. CAT activity calculated as reduced H_2_O_2_ in milligram to the negative one protein minute to the negative one was measured by determining the reduction in absorbance at 240 nm. APX was determined according to the procedure described by Dzung et al. [69]. The APX activity was calculated by measuring the ascorbate oxidation rate at 290 nm. The GR activity was measured according to Mandhania et al. [70]. The GR activity was assessed by tracking NADPH oxidation by observing the reduction in the absorbance at 340 nm over 3 min. Dehydroascorbate reductase (DHAR) activity was estimated according to Mishra, Bhoomika, and Dubey [44] by assaying the decrease in DHA in the presence of glutathione at 265 nm. DHAR activity is defined as micromole DHA reduced milligram to the negative one protein minute to the negative one. The peroxidase (POD) activity in milk thistle leaves was determined as described by Thomas et al. [71]. POD was measured using guaiacol as the substrate. The crude enzyme extract was prepared, as mentioned above, with SOD and CAT. The absorbance was then measured at 436 nm. POD activity was expressed in A436 Units milligram to the negative one·DM·minute to the negative one.

### 4.9. Determination of mRNA Levels

For Rt-PCR and qPCR, total RNA was isolated from leaf samples of milk thistle plants using an RNeasy Mini Kit (Qiagen, Valencia, CA, USA) according to the manufacturer’s recommendations. The RNA samples were treated with RNase-free DNAse (Qiagen) to remove any contaminant of genomic DNA. To synthesize the first cDNA strand, the RevertAid H Minus First Strand cDNA Synthesis Kit (Fermentas GmbH, St. Leon-Rot, Germany) was used. All primer names and sequences used for semi-quantitative and qPCR are listed in Table S1. Semi-quantitative RT-PCR of all investigated genes is presented in Figure S1. iCycler Thermal Cycler (Bio-Rad, Irvine, CA, USA) was used to perform the qPCR using the iQ SYBR Green Supermix (Bio-Rad) according to the manufacturer’s instructions. The actin housekeeping gene was used as an endogenous control for qPCR data normalization. The PCR efficiencies of each reaction were calculated according to the method described by Ruijter et al. [72] using LinRegPCR V. 11 software (Academic Medical Centre, Amsterdam, the Netherlands). For an accurate analysis, relative quantitative methods assume that the target and endogenous genes amplify with similar efficiency. The comparative C_T_ method (2^−ΔΔCT^) was used to calculate the expression level derived from the threshold cycles using the equation of Pfaffl [73]. 

### 4.10. Flavonolignans Analysis

The flavonolignans were extracted from the milk thistle dried seeds after defatting with Hexane overnight by steeping on a magnetic stirrer. Flavonolignans were then extracted with 80% methanol overnight by steeping on a magnetic stirrer. Extracts were filtered through a filter paper (Whatman No. 1, GE Healthcare Bio-Sciences, Pittsburgh, PA, USA) and lyophilized. The lyophilized samples were kept at −20°C until analysis. Silymarin was identified and measured using high-performance liquid chromatography (HPLC) technique, as described by Quaglia, et al. [74].

### 4.11. Alignment of Sequences and Construction of Phylogenetic Tree

Genbank databases searches for homologies to chalcone synthase (*CHS1*, *CHS2,* and *CHS3*) of milk thistle were accomplished using FASTA and WU-BLAST2 based on the basic local alignment search tool algorithm programs. Amino acid sequences were aligned using CLUSTALW 2.1 [75] software with default parameters. The phylogenetic trees were constructed using the maximum likelihood (ML) algorithm incorporated in MEGA ver. 5 program [76]. Bootstrap analyses with 500 repeats were achieved to assess the robustness of the branches.

### 4.12. Statistical Analyses

All data were analyzed by one-way ANOVA [77] using the MSTAT-C statistical package [78]. Means were compared and separated using Fisher’s least significant difference (LSD) test (*p* < 0.05) when there were significant treatment effects.

## Figures and Tables

**Table 1:** Changes in the flavonolignans in milk thistle seed (mg·g^−1^ DM) under mild (75%) and severe (50%) levels of drought stress compared with the well-watered (100%) condition.

Field Capacity	Silymarin	Silybin	Isosilybin	Silydianin	Taxifolin	Silychristin
100%	18.77 c	11.44 c	3.36 b	1.85 b	0.83 c	1.29 b
75%	19.91 b	12.34 b	3.39 b	1.82 b	1.04 b	1.34 b
50%	23.76 a	13.87 a	4.30 a	2.46 a	1.38 a	1.75 a
LSD * 5%	0.776	0.554	0.464	0.287	0.167	0.214

* LSD = Least Significant Difference test. Means in the same column followed by different letters are significantly different based on Fisher’s least significant difference (LSD) test (*p* < 0.05).

## Supplementary Materials

The following are available online at https://www.mdpi.com/2223-7747/8/12/611/s1, Figure S1: Semi-quantitative RT-PCR of antioxidant enzymes genes, *DAHR*, *GR*, *APX*, *SOD*, and *CAT*, and expression of *1-Cys-Prx* and *PrxQ*, additionally, expression of chalcone synthase genes (*CHS1*, *CHS2*, and *CHS3*) of milk thistle (*Silybum marianum* L.), Figure S2: Schematic presentation of flavonolignan biosynthetic pathway Table S1: Primers sequences for semi-quantitative and quantitative RT-PCR.
