# Supplementary material for: The Impact of Drought Stress on Antioxidant Responses and Accumulation of Flavonolignans in Milk Thistle (Silybum marianum (L.) Gaertn)
Source: Plants (Basel). 2019 Dec 16;8(12):611. doi: 10.3390/plants8120611 (PMC6963737; doi:10.3390/plants8120611)
Supplement: Supplementary file 1 [file plants-08-00611-s001.pdf]

Supplementary Materials

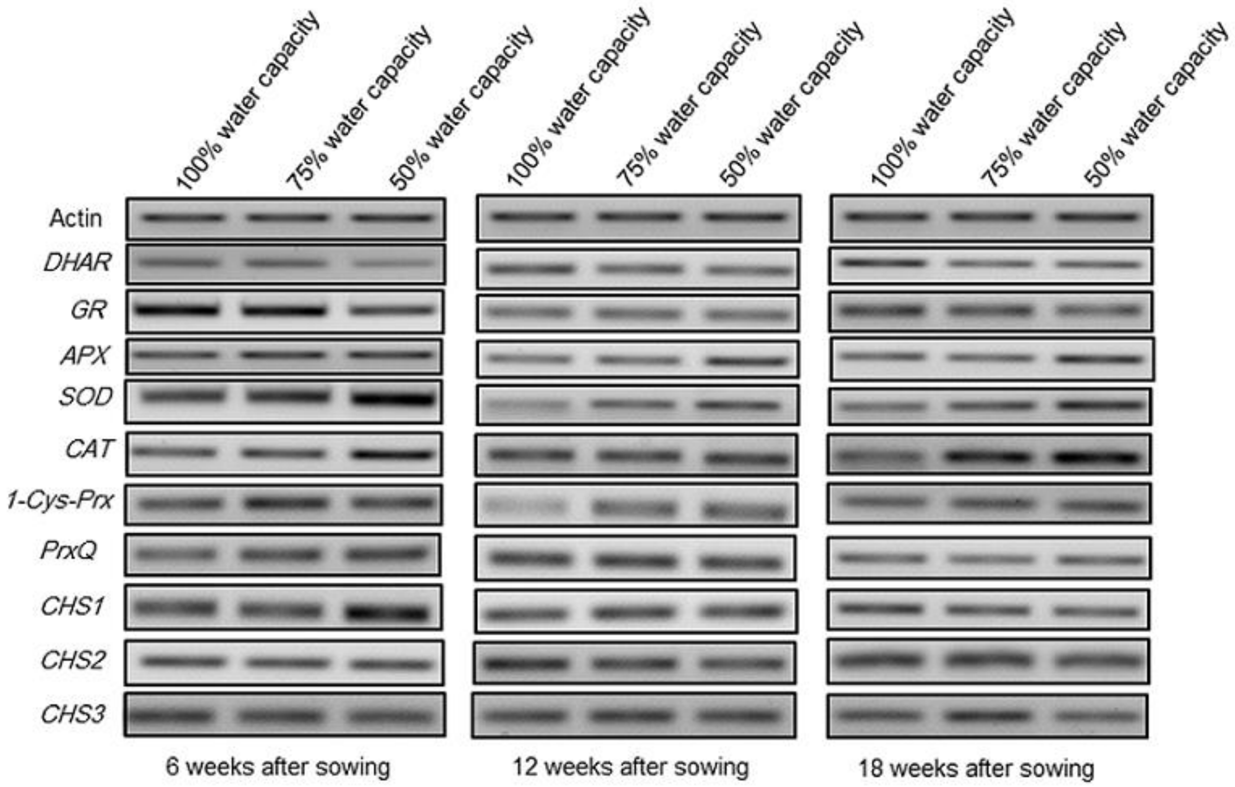

Figure S1. Semi-quantitative RT-PCR of antioxidant enzymes genes *DAHR*, *GR*, *APX*, *SOD*, and *CAT*, and expression of *1-Cys-Prx* and *PrxQ*, additionally, expression of chalcone synthase genes (*CHS1*, *CHS2*, and *CHS3*) from cDNA of milk thistle (*Silybum marianum* L.) leaves treatments with 100% water capacity, 75% water capacity and 50% water capacity at different time points. Samples standardized to actin. RT-PCR product were separated on agarose gels (1.5%) and visualized by ethidium bromide stain. *DHAR* dehydroascorbate reductase; *SOD* superoxide dismutase; *CAT* catalase; *APX* ascorbate peroxidase; *GR* glutathione reductase; *1-Cys-Prx* 1-cysteine peroxiredoxin; *PrxQ* peroxiredoxin Q.

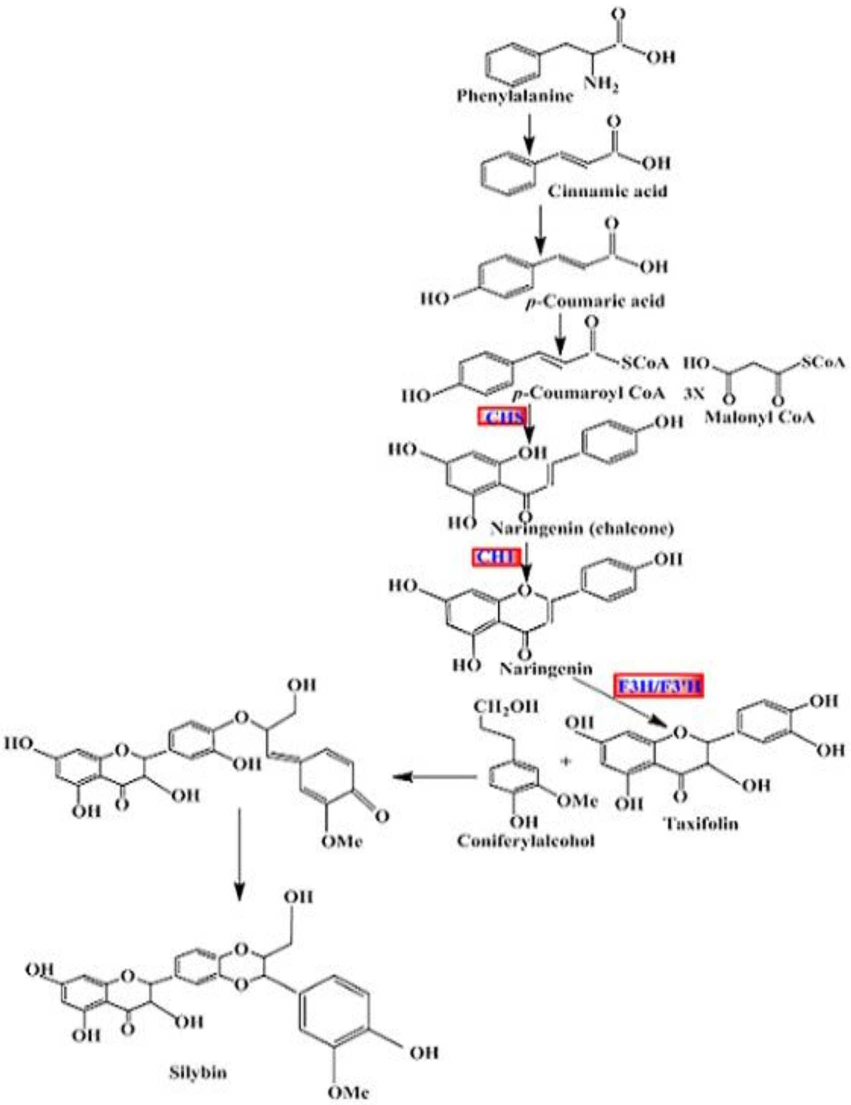

Figure S2. Schematic presentation of flavonolignan biosynthetic pathway. CHS, chalcone synthase; CHI, chalcone isomerase; F3H, flavanone 3-hydroxylase; F3'H, flavonol 3'-hydroxylase). The end product of the pathway is silybin.

Table S1. Primers sequences for semi quantitative and quantitative RT-PCR of the drought-related genes in milk thistle (*Silybum marianum* L.).

Gene name	Reference Sequence	5′ - 3′ primer sequence	T_A
Actin	AB181991	CTCTGACAATTTCCCGCTCA<br>ACACGCTTCCTCATGCTATCC	58 ℃
CHS1	JN182805.1	GTGTATACCAAGCCGATTATCCA<br>GTGTGAATACATACACATGCGCT	58 ℃
CHS2	JN182806.1	GTGCCTGCGTGTTGTTCATC<br>CTGTGAAGGACCACCGTCTC	58 ℃
CHS3	JN182807.1	GGGAGGAAGGGCTCACATTT<br>ATGCCCAATGGAAAAACCGC	58 ℃
DHAR	AY074784	TCAAGAACGTGACAAAGGTGG<br>TAACGGTGGTGATGGCAAAT	58 ℃
SOD	MG893090.1	TTCGCCATGCTGGTGATCTT<br>CATGGACAACTACGGCCCTT	58 ℃
CAT	GU984379	GGCTGCTTGAAGTTGTTCTCCT<br>CTGCTAGTACCTCCTGATCCGTT	58 ℃
APX	KU747079.1	TGGCCTGCTCTTCCTCTAGT<br>CATGCCACGCTAATCGAAGC	58 ℃
GR	KX828561.1	CAACGCGCTTTGGTAACTCC<br>GGGCCCTAATGAAGTGGAGG	58 ℃
1-Cys-Prx	AY304482	AATGGACCAAGGACATCGAG<br>GACGGGTACAGGAAGCTCAG	58 ℃
PrxQ	AY789643	ACTTCACGCTCAAGGACCAG<br>CCGCCTTCTTGTACTTCTCG	58 ℃
